# Machine Learning-Driven Prediction of Intensive Care Units Mortality and Length of Stay: A 11-Year Retrospective Study in Hong Kong Public Hospitals

**DOI:** 10.1007/s10916-026-02355-8

**Published:** 2026-03-10

**Authors:** Ying Zhao, Xincheng Shu, Chi-Sing Leung, Eric W. M. Wong, Qi Xuan, Kar-Lung Lee, Anne Leung, Lowell Ling, Hoi-Ping Shum, Wing-Lun Wan, Pauline Yeung Ng, Tsz-Kin Yim, Wai-Ming Tang, Kenny King-Chung Chan, Gavin Joynt

**Affiliations:** 1 Department of Electrical Engineering, City University of Hong Kong, Kowloon, Hong Kong SAR, China; 2 Institute of Cyberspace Security, Zhejiang University of Technology, Hangzhou, 310023 China; 3 Binjiang Institute of Artificial Intelligence, Zhejiang University of Technology, Hangzhou, 310056 China; 4 Department of Intensive Care, United Christian Hospital, 130 Hip Wo Street, Kwun Tong, Kowloon Hong Kong SAR, China; 5 Intensive Care Unit, Queen Elizabeth Hospital, 30 Gascoigne Road, Kowloon, Hong Kong SAR, China; 6 Department of Anaesthesia and Intensive Care, The Chinese University of Hong Kong, Shatin, New Territories Hong Kong SAR, China; 7 Department of Intensive Care, Pamela Youde Nethersole Eastern Hospital, 3 Lok Man Road, Chai Wan, Hong Kong SAR, China; 8 Department of Intensive Care, Yan Chai Hospital, 7-11 Yan Chai Street, Tsuen Wan, New Territories Hong Kong SAR, China; 9 Critical Care Medicine Unit, The University of Hong Kong, Pokfulam, Hong Kong SAR, China; 10 Department of Intensive Care, Tuen Mun Hospital, 23 Tsing Chung Koon Road, Tuen Mun, Hong Kong SAR, China

**Keywords:** ICU, Machine learning, Mortality prediction, Length of stay prediction, Model interpretability

## Abstract

This study aims to develop a machine learning (ML)-based pipeline to predict intensive care unit (ICU) mortality and length of stay (LOS). A dataset including 140,904 ICU admissions was collected from 15 public hospitals in Hong Kong over an 11-year period. The proposed pipeline deployed a suite of ML models to predict mortality and LOS. The performance of ML models was compared with the Acute Physiology and Chronic Health Evaluation (APACHE) systems on the collected dataset using five-fold cross-validation. Among all involved models, the Gradient Boosting with Categorical Features (CatBoost) achieved the highest area under the receiver operating characteristic curve (AUROC) of 0.9070 as well as the lowest Brier score of 0.0827 for mortality prediction and the lowest Mean Absolute Error (MAE) of 2.6364 for LOS prediction. The SHapley Additive exPlanations (SHAP) analysis conducted on CatBoost revealed that age, Glasgow Coma Scale (GCS) and urine output were the top-three important features for mortality prediction, whereas the top-three important features for LOS prediction were creatinine level, and the indicators for whether the lowest and highest respiratory rates were ventilator-measured. We further performed temporal validation and an in-depth analysis of CatBoost’s predictive performance across subsets grouped by age and hospital. Our results demonstrate that the proposed pipeline mitigates the overestimation of mortality predictions from APACHE systems in Hong Kong. Besides, the proposed predictive ML-based pipeline offers a transferable framework for researchers to develop models tailored to their local medical environments.

## Introduction

Intensive care unit (ICU) resources are under increasing strain globally. The resource shortage was amplified when major pandemic events occurred [1, 2]. Hence, an automated method for predicting ICU mortality and length of stay (LOS) is urgently needed [3–8]. These predictions can assist decision-making regarding the allocation of resources, quality of care benchmarking or evaluation by comparing predicted with actual outcomes over time (within and between units), standardizing populations into risk categories for research, and evaluating healthcare workloads [9].

Existing prognostic models, such as the Acute Physiology and Chronic Health Evaluation (APACHE) scoring systems [10–12], Sequential Organ Failure Assessment (SOFA) scoring systems [13], and the Simplified Acute Physiology Score (SAPS) [14–17] have long been used for predicting ICU outcomes. In Hong Kong, APACHE IV has been adopted by the Hospital Authority (HA) for ICU monitoring since 2007 [18]. However, owing to the advancements in clinical environments, APACHE IV has been shown to suffer from overestimating mortality rates for ICU patients in Hong Kong from 2008 to 2018 [19]. The above traditional scoring methods may struggle to adapt to evolving clinical practice and varying patient populations [20, 21].

Recent studies have demonstrated the effectiveness of machine learning (ML) models in prognosis and outcome prediction [22–27]. However, some existing ML studies on ICU outcomes are limited by relatively small dataset sizes exemplified by work from Sonu et al. [28] (3,597 patients), Dan et al. [29] (733 patients), and Huo et al. [30] (1,242 patients). Some studies focus on specific disease contexts like COVID-19 [28, 29, 31], while some lack multi-center validation [21, 32]. Although studies incorporating larger-scale databases have emerged, they often present other limitations. For instance, Arjun et al. developed ML models to predict LOS after surgery, but their work lacked interpretability frameworks [33]. Safaei et al. [34] proposed an ML framework to predict ICU mortality for twelve distinct disease groups using the eICU Collaborative Research Database (eICU). However, their work did not include LOS prediction, lacked temporal validation for model stability, and did not conduct subgroup analysis for performance across diverse age cohorts or hospitals. Furthermore, some larger-scale studies [34–36] rely on public databases collected from hospitals across the United States (e.g., the Medical Information Mart for Intensive Care IV, eICU), which may not accurately reflect local clinical practice or patient characteristics in Asian regions.

Given these persistent limitations, where existing works are often constrained by small data sizes or lack comprehensive analyses such as subgroup analysis, temporal validation, and interpretability frameworks, we developed a transferable ML-based pipeline providing comprehensive analyses for simultaneous ICU mortality and LOS prediction based on an extensive dataset collected from 15 public hospitals over an 11-year period in Hong Kong. Our contributions are listed as follows:

1. The proposed pipeline deploys seven ML models to predict mortality and LOS using five-fold cross-validation. To the best of our knowledge, none of the existing large-scale, multi-center ICU predictive models are particularly designed to be compatible with ICU clinical practice in Hong Kong, simultaneously addressing both ICU mortality and LOS predictions. This pipeline demonstrated superior predictive performance for both tasks compared to APACHE systems. This directly addresses the persistent overestimation of ICU mortality by APACHE IV in Hong Kong.
2. Through Shapley Additive exPlanations (SHAP) [37] analysis on CatBoost, the best performing ML model, we identified key clinical features influencing predictions. This feature importance analysis provides actionable insights to enhance clinical monitoring and support decision-making in ICUs.
3. We also performed temporal validation and subgroup analysis (based on age and hospital) on CatBoost. These analyses revealed how model performance varies across different patient age groups and institutional settings, and provided critical insights into the model’s stability over time when faced with evolving clinical data.
4. The proposed pipeline with explainable frameworks and in-depth analyses offers practical guidance for the deployment and adaptation of ML models in diverse clinical environments, enabling other researchers to develop and implement predictive models tailored to their unique local patient populations and healthcare systems.

## Methods

### Datasets

**Fig. 1:**
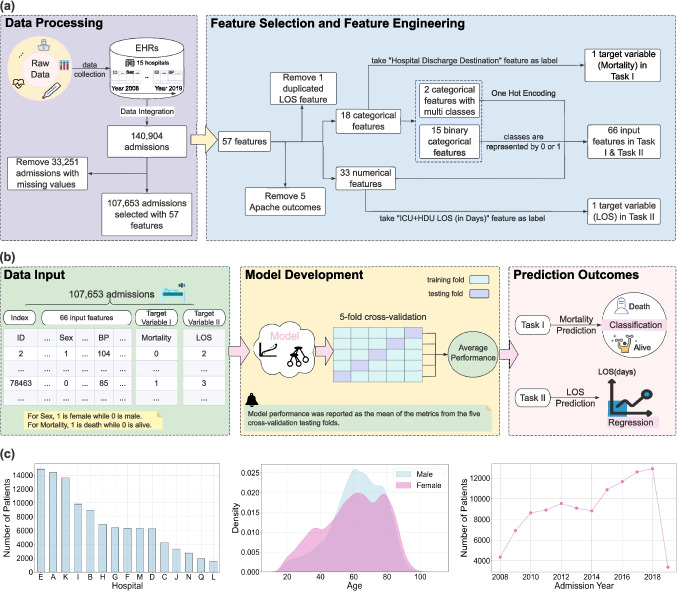
Overview of the study framework. (**a**) describes the data processing, feature selection and feature engineering; (**b**) describes how to develop and evaluate the model in our pipeline. (**a**) and (**b**) illustrate the complete ML-based pipeline proposed in this study. (**c**) shows the distribution of patients by hospital group and admission year, and patients density across different age groups

We collected data from the Clinical Data Analysis and Reporting System (CDARS) of HA, the Census and Statistics Department of the Government of Hong Kong, and the Hospital Authority’s Annual Statistical Reports by the APACHE IV system. The dataset comprised 140,904 ICU admissions (age $\ge 16$) across 15 public hospitals in Hong Kong from 1 April 2008 to 31 March 2019. To ensure data integrity, 33,251 records with missing values were excluded from this study, as shown in Fig. 1(a). In total, 107,653 ICU admission records were retained for model development.

### Feature and Target Variables

The remaining ICU admission records contained 57 features, including 18 categorical and 39 numerical variables. As illustrated in Fig. 1(a), feature selection was first performed on the admission records by excluding the five APACHE outcome variables and one repeated LOS feature from the feature set. Then, binary categorical variables were converted into binary values (0 or 1), while multiclass categorical variables were transformed into binary representations using one-hot encoding, where a value of 1 indicates the presence of a category and 0 indicates its absence. As a result, the processed dataset consisted of 66 numerical input features and two target variables (Hospital Discharge Destination and ICU+HDU LOS (in days)) representing the ground truth of ICU mortality and LOS predictions.

The input features were categorized into seven groups: (1) basic demographics (e.g., age, sex); (2) comorbidities such as cardiovascular and respiratory diseases; (3) diagnosis on admission; (4) ICU admission sources; (5) ICU admission types; (6) worst vital signs (first 24 hours in ICUs); and (7) laboratory findings (first 24 hours in ICUs). Data were collected from each patient’s first ICU admission, with the earliest laboratory results selected for analysis. Descriptive statistics of these features are provided in Table 1. The same feature set was used for both the baseline models and the ML models.

**Table 1:** Statistics of basic patient characteristics, ICU admission reasons, and laboratory information used across different cohorts. Data are presented as median (IQR) for numerical variables and $n (\%)$ for categorical variables. P-values are calculated using Student’s t-test

Variables	Total	Dead Cohort	Alive Cohort	P-value
	(n=107,653)	(n=18,648)	(n=89,005)	
Demographic data				
Age, median(IQR)	63.0 (51.00–74.00)	69.0 (58.00–79.00)	61.0 (49.00–73.00)	<0.001
Female, n(%)	41679 (38.72%)	6187 (33.18%)	35492 (39.88%)	<0.001
Comorbidity				
Cardiovascular, n(%)	308 (0.29%)	100 (0.54%)	208 (0.23%)	<0.001
Respiratory, n(%)	1727 (1.6%)	439 (2.35%)	1288 (1.45%)	<0.001
Renal, n(%)	4097 (3.81%)	1133 (6.08%)	2964 (3.33%)	<0.001
Hepatic failure, n(%)	1222 (1.14%)	373 (2.0%)	849 (0.95%)	<0.001
Cirrhosis, n(%)	1827 (1.7%)	484 (2.6%)	1343 (1.51%)	<0.001
AIDS, n(%)	202 (0.19%)	60 (0.32%)	142 (0.16%)	<0.001
Lymphoma, n(%)	831 (0.77%)	383 (2.05%)	448 (0.5%)	<0.001
Metastases, n(%)	3299 (3.06%)	610 (3.27%)	2689 (3.02%)	0.071750
Leukaemia/ Myeloma, n(%)	945 (0.88%)	448 (2.4%)	497 (0.56%)	<0.001
Immunosupression, n(%)	5257 (4.88%)	1539 (8.25%)	3718 (4.18%)	<0.001
Diagnosis on admission				
Cardiovascular, n(%)	14684 (13.64%)	3361 (18.02%)	11323 (12.72%)	<0.001
Gastrointestinal, n(%)	23412 (21.75%)	3626 (19.44%)	19786 (22.23%)	<0.001
Gynaecological, n(%)	2061 (1.91%)	17 (0.09%)	2044 (2.3%)	<0.001
Metabolic, n(%)	8945 (8.31%)	385 (2.06%)	8560 (9.62%)	<0.001
Neurological, n(%)	14542 (13.51%)	1842 (9.88%)	12700 (14.27%)	<0.001
Renal/Genitourinary, n(%)	6269 (5.82%)	803 (4.31%)	5466 (6.14%)	<0.001
Respiratory, n(%)	15267 (14.18%)	2343 (12.56%)	12924 (14.52%)	<0.001
Sepsis, n(%)	16907 (15.71%)	5582 (29.93%)	11325 (12.72%)	<0.001
Trauma, n(%)	5566 (5.17%)	689 (3.69%)	4877 (5.48%)	<0.001
ICU Admission source				
Emergency Department, n(%)	18658 (17.33%)	2882 (15.45%)	15776 (17.72%)	<0.001
General ward, n(%)	38612 (35.87%)	10120 (54.27%)	28492 (32.01%)	<0.001
OT/Recovery, n(%)	47942 (44.53%)	4790 (25.69%)	43152 (48.48%)	<0.001
Other CCU in same hospital, n(%)	885 (0.82%)	356 (1.91%)	529 (0.59%)	<0.001
Other HDU in same hospital, n(%)	877 (0.81%)	304 (1.63%)	573 (0.64%)	<0.001
Other ICU in same hospital, n(%)	41 (0.04%)	16 (0.09%)	25 (0.03%)	<0.001
Other hospital CCU, n(%)	77 (0.07%)	31 (0.17%)	46 (0.05%)	<0.001
Other hospital HDU, n(%)	20 (0.02%)	8 (0.04%)	12 (0.01%)	0.007360
Other hospital ICU, n(%)	172 (0.16%)	39 (0.21%)	133 (0.15%)	0.063418
Other hospital general ward, n(%)	369 (0.34%)	102 (0.55%)	267 (0.3%)	<0.001
ICU Admission type				
Elective, n(%)	25199 (23.41%)	1023 (5.49%)	24176 (27.16%)	<0.001
Emergency, n(%)	82454 (76.59%)	17625 (94.51%)	64829 (72.84%)	<0.001
Worst vital signs (in first 24 hours of ICU)				
Glasgow coma scale, median(IQR)	15.0 (12.00–15.00)	11.0 (3.00–15.00)	15.0 (14.00–15.00)	<0.001
Core temperature - high, median(IQR)	37.7 (37.20–38.30)	37.7 (36.90–38.40)	37.7 (37.20–38.30)	<0.001
Core temperature - low, median(IQR)	36.3 (35.80–36.80)	36.0 (35.00–36.50)	36.3 (36.00–36.80)	<0.001
Heart rate - high (/min), median(IQR)	109.0 (93.00–126.00)	122.0 (105.00–140.00)	106.0 (91.00–122.00)	<0.001
Heart rate - low (/min), median(IQR)	73.0 (62.00–85.00)	77.0 (60.00–92.00)	72.0 (62.00–84.00)	<0.001
Highest Respiratory Rate (/min), median(IQR)	26.0 (22.00–31.00)	29.0 (24.00–35.00)	26.0 (22.00–30.00)	<0.001
Lowest Respiratory Rate (/min), median(IQR)	13.0 (11.00–15.00)	14.0 (12.00–16.00)	12.0 (11.00–15.00)	<0.001
Mean BP - high (mmHg), median(IQR)	106.0 (95.00–119.00)	102.0 (90.00–116.00)	106.0 (95.00–119.00)	<0.001
Mean BP - low (mmHg), median(IQR)	65.0 (58.00–75.00)	57.0 (47.00–66.00)	67.0 (60.00–76.00)	<0.001
Urine (ml/24 hrs), median(IQR)	1355.0 (855.00–2027.00)	808.0 (200.00–1530.00)	1440.0 (970.00–2105.00)	<0.001
Laboratory findings (in first 24 hours of ICU)				
Na+ - high (mmol/L), median(IQR)	140.0 (137.20–143.00)	141.0 (137.00–145.00)	140.0 (137.40–142.80)	<0.001
Na+ - low (mmol/L), median(IQR)	137.2 (134.00–140.00)	137.0 (133.00–141.00)	137.3 (134.69–140.00)	<0.001
K+ - high (mmol/L), median(IQR)	4.14 (3.80–4.60)	4.4 (3.90–5.03)	4.1 (3.80–4.55)	<0.001
K+ - low (mmol/L), median(IQR)	3.6 (3.30–4.00)	3.6 (3.20–4.10)	3.6 (3.30–3.95)	<0.001
Creat - high (mmol/L), median(IQR)	97.0 (69.00–177.00)	171.0 (99.00–310.00)	90.0 (67.00–148.00)	<0.001
Albumin - low (g/L), median(IQR)	27.2 (22.00–33.00)	22.0 (17.00–28.00)	28.2 (23.00–33.60)	<0.001
Bilirubin - high (umol/L), median(IQR)	14.84 (9.40–24.00)	17.6 (10.70–33.20)	14.06 (9.10–23.00)	<0.001
WCC - low (10^^^9/L), median(IQR)	10.5 (7.55–14.00)	10.4 (6.20–15.09)	10.5 (7.71–13.80)	<0.001
WCC - high (10^^^9/L), median(IQR)	13.8 (10.20–18.40)	14.43 (9.51–20.38)	13.7 (10.30–18.08)	<0.001
Hb - high (g/L), median(IQR)	11.5 (10.00–13.20)	10.7 (9.30–12.60)	11.6 (10.10–13.30)	<0.001
Hb - low (g/L), median(IQR)	10.2 (8.50–12.00)	9.1 (7.40–11.00)	10.5 (8.80–12.10)	<0.001
Hct - high, median(IQR)	0.34 (0.30–0.39)	0.32 (0.28–0.38)	0.35 (0.30–0.39)	<0.001
Hct - low, median(IQR)	0.31 (0.26–0.36)	0.27 (0.23–0.33)	0.31 (0.27–0.36)	<0.001
Platelet - high (10^^^9/L), median(IQR)	205.0 (148.00–271.00)	177.0 (113.00–258.00)	210.0 (155.00–273.00)	<0.001
Platelet - low (10^^^9/L), median(IQR)	177.0 (121.00–236.00)	138.0 (74.00–213.00)	182.0 (131.00–239.00)	<0.001
Glucose - high (mmol/L), median(IQR)	9.2 (7.50–12.10)	10.1 (7.90–13.80)	9.1 (7.40–11.80)	<0.001
Glucose - low (mmol/L), median(IQR)	5.8 (4.80–7.10)	5.5 (4.07–7.10)	5.9 (4.90–7.10)	<0.001
1st pH taken in ICUs, median(IQR)	7.37 (7.29–7.42)	7.31 (7.19–7.41)	7.37 (7.31–7.42)	<0.001
1st PaCO2 taken in ICUs (mmHg), median(IQR)	37.12 (31.50–43.27)	37.2 (30.00–47.33)	37.12 (31.65–42.75)	<0.001
1st PaO2 taken in ICUs (mmHg), median(IQR)	118.5 (87.01–165.97)	113.57 (80.62–173.26)	119.25 (88.51–165.00)	<0.001
FiO2 when 1st PaO2 is checked, median(IQR)	0.4 (0.25–0.50)	0.5 (0.40–1.00)	0.35 (0.25–0.50)	<0.001
Highest Respiratory Rate On Ventilator, n(%)	42340 (39.33%)	12676 (67.98%)	29664 (33.33%)	<0.001
Lowest Respiratory Rate On Ventilator, n(%)	47845 (44.44%)	13447 (72.11%)	34398 (38.65%)	<0.001
1st Arterial/Vensus_A, n(%)	105729 (98.21%)	18362 (98.47%)	87367 (98.16%)	0.004052

### Study Design

This study was approved by The Joint Chinese University of Hong Kong–New Territories East Cluster Clinical Research Ethics Committee (2020.078) with a waiver of informed consent and the local ethics committees of each participating ICU. Figure 1(a) and (b) depict the overall workflow of the proposed ML-based pipeline. We aimed to develop predictive models for two tasks:

- **Task I:** Predicting ICU mortality, i.e., identifying whether a patient will die after receiving ICU treatment.
- **Task II:** Predicting ICU LOS, i.e., estimating how long patients will stay in the ICU.

Five-fold cross-validation was applied during model development and validation to perform a fair comparison with all models involved in this study. Specifically, the entire dataset was randomly partitioned into five equally sized subsets (namely folds). For each candidate model, the cross-validation procedure was repeated five times. In each round, one distinct fold served as the hold-out testing set, while the remaining four folds were used for model training. The above approach guaranteed that all samples in our dataset were tested on each model one time, thereby mitigating sampling bias inherent in a single train-test split.

### Main Models

The proposed pipeline consists of two task-specific and five jointly utilized ML models, in which Logistic Regression (LR) [38] and Least Absolute Shrinkage and Selection Operator (LASSO) [39] are dedicated to predict ICU mortality (namely Task I) and LOS (namely Task II), respectively. Our model selection was designed to compare linear and non-linear models against the APACHE benchmarks. Given that APACHE IV, which was developed using multivariable LR is not publicly available for external training [11], we employed LR for Task I to serve as a transparent and directly comparable clinical baseline. For Task II, LASSO was chosen for its embedded feature selection capability, which is valuable in our moderately high-dimensional setting (66 features). Random Forest (RF) [40], Gradient Boosting Decision Tree (GBDT) [41], LightGBM [42], Extreme Gradient Boosting (XGBoost) [43], and Gradient Boosting with Categorical Features (CatBoost) [44] are employed on both tasks. All of the five shared ML models are ensemble models and can handle non-linear relationships as well as interactions among variables.

### CatBoost Model

CatBoost is a gradient boosting framework based on decision tree specifically designed to handle both numerical and categorical features, making it particularly effective for tabular data and a versatile choice for a wide range of ML applications [44]. The core of the CatBoost algorithm is the gradient boosting procedure [45], which iteratively constructs an ensemble of weak learners (decision trees) to optimize a given loss function. For a given input *x*, the CatBoost algorithm builds *T* trees, with the $t^{\text {th}}$ tree generating a score $h_t(x)$. The final predicted score *F*(*x*) is obtained by summing the weighted scores of all trees, expressed as follows:

1 $$\begin{aligned} F(x) = \sum _{t=1}^{T} \alpha _t h_t(x) \end{aligned}$$

In our experimental settings, we utilized the catboost.CatBoostClassifier and CatBoostRegressor implementations from the CatBoost Python library to develop the models for the two tasks, respectively.

### SHAP

The SHAP method is a powerful framework for interpreting ML models, providing insights into how individual features contribute to model predictions [37]. In our study, we utilized the shap.TreeExplainer to interpret the predictions made by the CatBoost model. For Task I, positive SHAP values indicate a higher risk of mortality, while negative values suggest a lower risk. Similarly, for Task II, positive SHAP values correspond to longer ICU stays, while negative values suggest a contribution towards a shorter one. By analyzing the SHAP values, we identified key features influencing the predictions, offering clinically meaningful insights.

### Evaluation Metrics

For Task I, we utilized two key metrics: the Area Under the Receiver Operating Characteristic Curve (AUROC) and the Area Under the Precision-Recall Curve (AUPRC). AUROC provides the overall classification ability, while AUPRC focuses on model performance for the rare positive class (Death). Calibration performance was evaluated using calibration curves [46] and the Brier score [47].

For Task II, performance was assessed using four metrics: Mean Absolute Error (MAE), Mean Square Error (MSE), Mean Absolute Percentage Error (MAPE) and Coefficient of Determination ($R^{2}$). Smaller MAE, MSE, and MAPE values indicate better model performance, while an $R^{2}$ value closer to 1 signifies a better fit of the model to the data.

## Results

### Mortality Prediction

**Fig. 2:**
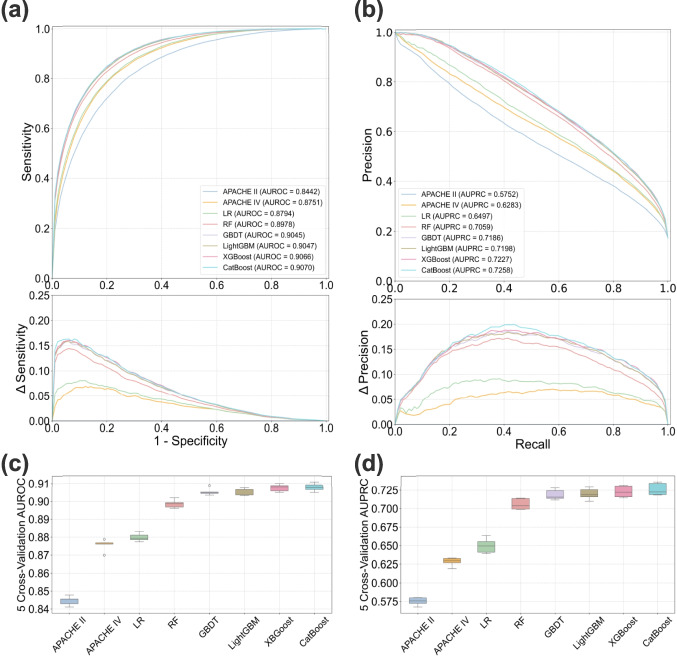
Performance comparison of all models for mortality prediction. (**a**) and (**b**) show the comparison of AUROC and AUPRC across all models, with lower sub-panels illustrating differences in sensitivity and precision relative to the baseline APACHE II. (**c**) and (**d**) present box plots of the AUROC and AUPRC results from five rounds of cross-validation for all models

Six ML models as described in “Main Models” and “CatBoost Model” sections were employed in Task I: LR, RF, GBDT, LightGBM, XGBoost, and CatBoost. Each model was trained and tested using 5-fold cross-validation, with the average performance and standard deviations reported. As summarized in Table 2 and Fig. 2, all ML models outperformed the benchmarks, APACHE II and IV. CatBoost achieved the highest overall performance, with an AUROC of 0.9070 and an AUPRC of 0.7258. This represents a significant improvement over the APACHE IV (AUROC 0.8751, AUPRC 0.6283).

**Table 2:** Performance comparison of different models on mortality prediction. Results are average performance from 5 testing folds, with standard deviations represented as ±. The bold values indicate the best performance

Model	AUROC	AUPRC
APACHE II	0.8442±0.0023	0.5752±0.0048
APACHE IV	0.8751±0.0030	0.6283±0.0051
LR	0.8794±0.0021	0.6499±0.0093
RF	0.8978±0.0021	0.7054±0.0074
GBDT	0.9045±0.0018	0.7180±0.0065
LightGBM	0.9047±0.0018	0.7198±0.0069
XGBoost	0.9066±0.0018	0.7227±0.0070
CatBoost	**0.9070**±**0.0022**	**0.7258**±**0.0077**

We observed the variability of AUROC and AUPRC metrics across the 5-fold cross-validation in Fig. 2(c) and (d). GBDT, LightGBM, and XGBoost showed the highest stability, with AUROC standard deviations of $\pm 0.0018$, while APACHE IV displayed a slightly higher variability ($\pm 0.0030$). Regarding AUPRC, LR exhibited the highest variability ($\pm 0.0093$). Although APACHE IV displayed the lowest standard deviation for AUPRC, its performance remained lower than that of ML models across all folds. These observations suggest that the ML models maintained reliable and stable performance across different data folds.

**Fig. 3:**
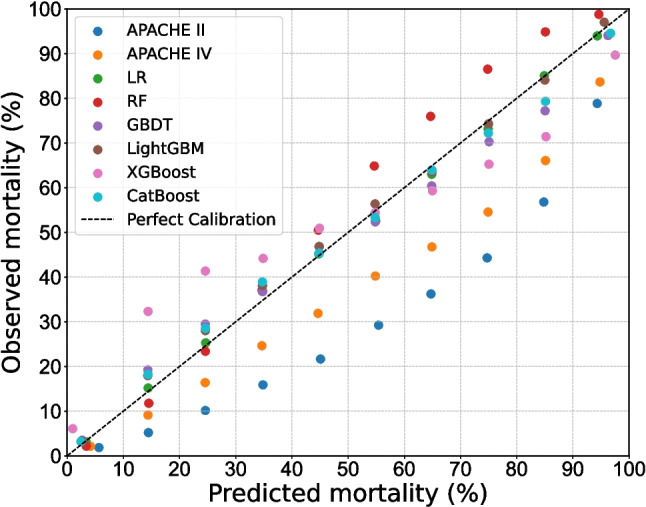
Calibration plots of all models for mortality prediction

Calibration analysis was conducted to evaluate the agreement between predicted probabilities and observed outcomes using calibration curves [19, 46] and the Brier score [47]. CatBoost, RF, and LightGBM displayed the closest alignment with perfect calibration in Fig. 3. This observation was confirmed by the Brier score, where CatBoost achieved the lowest Brier score of 0.0827, outperforming APACHE IV (0.1033) and APACHE II (0.1331). These findings demonstrate that ML models, particularly CatBoost, provide better calibrated predictions compared to benchmarks.

### LOS Prediction

**Fig. 4:**
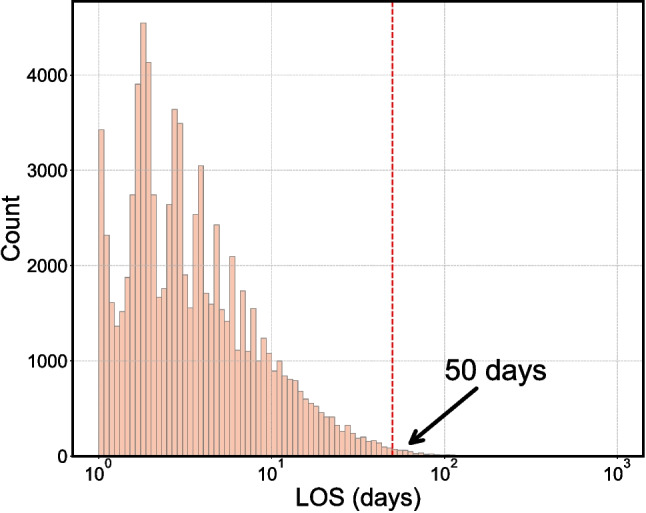
LOS (in days) instances distribution

The distribution of LOS in Task II is illustrated in Fig. 4, where the majority of patients (107,226, 99.6%) had an LOS ${\le }$50 days. To ensure comprehensive analysis, we developed six ML models, including LASSO, RF, GBDT, XGBoost, LightGBM, and CatBoost, across different LOS subgroups. The APACHE IV model served as the benchmark for comparison. Model performance was assessed using four metrics: MAE, MSE, MAPE, and ${R^{2}}$. Similar to Task I, each model was trained and tested using 5-fold cross-validation, with average performance metrics and standard deviations reported.

As shown in Table 3, CatBoost consistently outperformed all other models across all LOS subgroups and the full dataset. For patients with LOS ${\le }$ 50 days, CatBoost achieved an MAE of 2.3666, MSE of 26.0284, MAPE of 63.9421, and ${R^{2}}$ of 0.1859. This marked a significant improvement over the APACHE IV model, which had an MAE of 3.3205, MSE of 27.2087, MAPE of 176.8126, and ${R^{2}}$ of 0.1489. This superior performance trend held true for the full dataset and all other subgroups.

**Table 3:** Performance comparison of different models on LOS prediction across various subdatasets split by LOS. The bold values indicate the best performance

Model	MAE (± Std)	MSE (± Std)	MAPE (± Std)	${R^{2}}$ (± Std)
LOS $\le 50$ days				
APACHE IV	3.3205 ± 0.0120	27.2087 ± 0.2510	176.8126 ± 1.7253	0.1489 ± 0.0045
LASSO	2.7594 ± 0.0429	98.6175 ± 124.9065	85.9263 ± 0.3684	−2.1168 ± 3.9825
RF	2.4116 ± 0.0183	27.2419 ± 0.3703	67.1607 ± 0.4541	0.1480 ± 0.0037
GBDT	2.4646 ± 0.0191	27.7324 ± 0.3392	68.8940 ± 0.4053	0.1326 ± 0.0031
LightGBM	2.3960 ± 0.0197	26.4921 ± 0.3762	65.1254 ± 0.3206	0.1714 ± 0.0041
XGBoost	2.3846 ± 0.0201	26.2488 ± 0.3957	64.5926 ± 0.3290	0.1790 ± 0.0048
CatBoost	**2.3666** ± **0.0177**	**26.0284** ± **0.3683**	**63.9421** ± **0.2556**	**0.1859** ± **0.0041**
LOS $\le 100$ days				
APACHE IV	3.5071 ± 0.0189	39.0929 ± 0.8352	176.5105 ± 0.9484	0.1314 ± 0.0049
LASSO	2.9550 ± 0.0331	96.4366 ± 93.8766	86.9359 ± 0.7258	−1.1885 ± 2.1986
RF	2.6063 ± 0.0252	39.7552 ± 0.8475	68.1590 ± 0.5778	0.1167 ± 0.0033
GBDT	2.6603 ± 0.0265	40.3264 ± 0.8253	69.7397 ± 0.6362	0.1040 ± 0.0034
LightGBM	2.5912 ± 0.0222	38.8694 ± 0.7896	66.0008 ± 0.5593	0.1364 ± 0.0040
XGBoost	2.5796 ± 0.0219	38.5615 ± 0.7907	65.4780 ± 0.5208	0.1432 ± 0.0041
CatBoost	**2.5619** ± **0.0232**	**38.2977** ± **0.8916**	**64.7825** ± **0.6140**	**0.1491** ± **0.0047**
LOS $\le 200$ days				
APACHE IV	3.5646 ± 0.0375	46.6040 ± 1.9245	176.4700 ± 1.1196	0.1180 ± 0.0041
LASSO	3.0078 ± 0.0437	82.1776 ± 54.6141	87.0475 ± 0.6521	−0.5287 ± 0.9546
RF	2.6658 ± 0.0419	47.4923 ± 2.0313	68.3147 ± 0.5292	0.1012 ± 0.0050
GBDT	2.7206 ± 0.0416	48.0833 ± 2.0661	69.9911 ± 0.5482	0.0901 ± 0.0050
LightGBM	2.6499 ± 0.0417	46.5213 ± 2.0619	66.1805 ± 0.5003	0.1197 ± 0.0067
XGBoost	2.6372 ± 0.0411	46.1786 ± 2.0670	65.5646 ± 0.6402	0.1262 ± 0.0071
CatBoost	**2.6214** ± **0.0413**	**45.9646** ± **2.0420**	**65.0081** ± **0.5499**	**0.1302 ± 0.0069**
Full dataset				
APACHE IV	3.5796 ± 0.0321	53.3198 ± 9.4870	176.4663 ± 1.7600	0.1075 ± 0.0116
LASSO	3.0269 ± 0.0523	107.2908 ± 88.8471	87.0675 ± 0.5784	−0.8733 ± 1.6679
RF	2.6808 ± 0.0324	54.2661 ± 9.6395	68.3406 ± 0.4005	0.0916 ± 0.0114
GBDT	2.7345 ± 0.0319	54.8636 ± 9.6733	69.9681 ± 0.3730	0.0815 ± 0.0106
LightGBM	2.6673 ± 0.0324	53.3350 ± 9.5979	66.3518 ± 0.4258	0.1075 ± 0.0129
XGBoost	2.6538 ± 0.0301	52.9717 ± 9.5878	65.7378 ± 0.3806	0.1137 ± 0.0136
CatBoost	**2.6364** ± **0.0325**	**52.7217** ± **9.5995**	**65.1247** ± **0.4153**	**0.1180** ± **0.0144**

### Feature Analysis

**Fig. 5:**
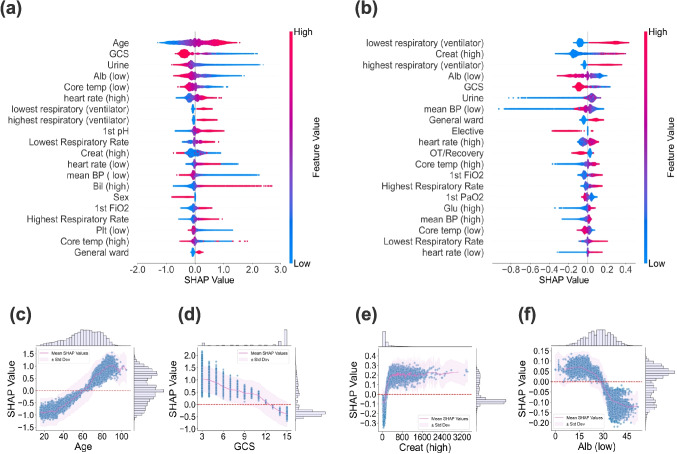
SHAP analysis of CatBoost for mortality and LOS. (**a**) and (**b**) present the top 20 most important features influencing mortality and LOS, respectively, as determined by SHAP values. Higher SHAP values indicate an increased probability of mortality and a longer LOS. Each dot represents an individual patient, with colors reflecting the magnitude of predictors: red indicates a higher feature value, while blue indicates a lower feature value. (**c**)-(**f**) show scatter plots of the relationships between individual features, i.e., age, GCS, Creat (high), and Alb (low) and their corresponding SHAP values for mortality and LOS respectively. The purple line and shaded area represent the mean and standard deviation of the regression line. Histograms on the right and top of each scatter plot illustrate the distributions of SHAP and feature values, respectively. High variance in SHAP values for a given feature suggests significant influence from other predictors

To interpret the predictions of the best performing model CatBoost, we employed SHAP analysis to identify the most influential features for both tasks, presented in Fig. 5.

For Task I, age emerged as the most significant feature, followed by Glasgow Coma Scale (GCS) and Urine (urine output) (Fig. 5(a)). For Task II, lowest respiratory (ventilator) (whether measuring the lowest respiratory rate on a ventilator), Creat (high) (high creatinine levels) and highest respiratory (ventilator) (whether measuring the highest respiratory rate on a ventilator) were identified as the most important features (Fig. 5(b)).

The detailed relationships between specific feature values, including age, GCS, Creat (high), and Alb (low) (low albumin levels) and their corresponding SHAP values are illustrated in Fig. 5(c)-(f). Each scatter plot displays the SHAP value distribution across feature values, indicating their contribution to prediction outcomes. Older age and lower GCS were strongly associated with increased mortality probability, while high Creat (high) and low Alb (low) would lead to a longer LOS.

### Subgroup Performance

We evaluated the performance of CatBoost across different subgroups based on age and hospital using five-fold cross-validation. All reported performance metrics represent the mean values obtained from five-fold cross-validation.

**Fig. 6:**
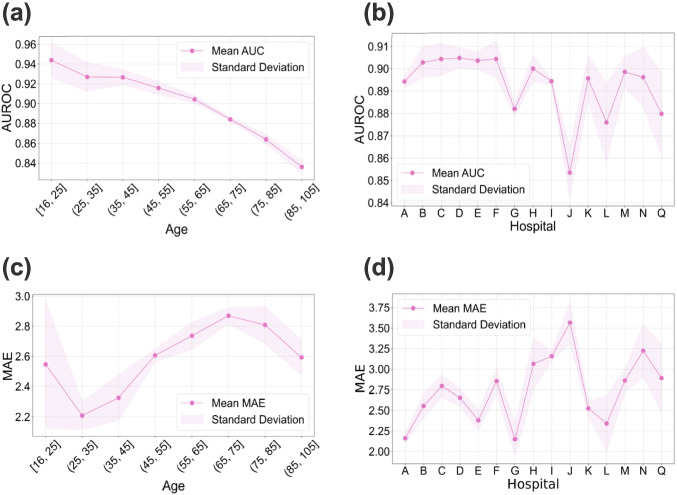
Performance of the CatBoost model in predicting mortality and LOS across different patient cohorts. (**a**) and (**b**) display AUROC for mortality prediction stratified by age groups and hospital groups respectively. For age and hospital groups, the model was trained on different cohorts split by age and hospital, with results obtained from 5-fold cross-validation. (**c**) and (**d**) display MAE for LOS prediction using the same dataset split

The analysis by age revealed distinct patterns for each prediction task. For mortality prediction, a clear inverse relationship was observed between age and model performance (Fig. 6(a)). CatBoost achieved its highest AUROC of 0.9438 in the youngest (16, 25] cohort, which systematically declined to its lowest point of 0.8362 in the oldest (85, 105] age group. In contrast, the model’s performance on LOS prediction followed a non-linear trend in relation to age (Fig. 6(c)). CatBoost performed best for younger adults, achieving its lowest MAE of 2.2086 in the (25, 35] age group. The prediction error then generally increased with age, peaking for the (65, 75] cohort (MAE = 2.8696).

We further assessed the model’s performance across different hospital groups to evaluate its transferability. For mortality prediction, CatBoost demonstrated high performance, with the AUROC ranging from a high of 0.9048 (Hospital D) to a low of 0.8535 (Hospital J) (Fig. 6(b)). For LOS prediction, the performance showed a slightly wider range (Fig. 6(d)). The MAE varied from a low of 2.1506 (Hospital G) to a high of 3.5655 (Hospital J). Importantly, the standard deviations of the MAE for each hospital were relatively small, indicating consistent performance within each clinical site.

### Temporal Validation

**Fig. 7:**
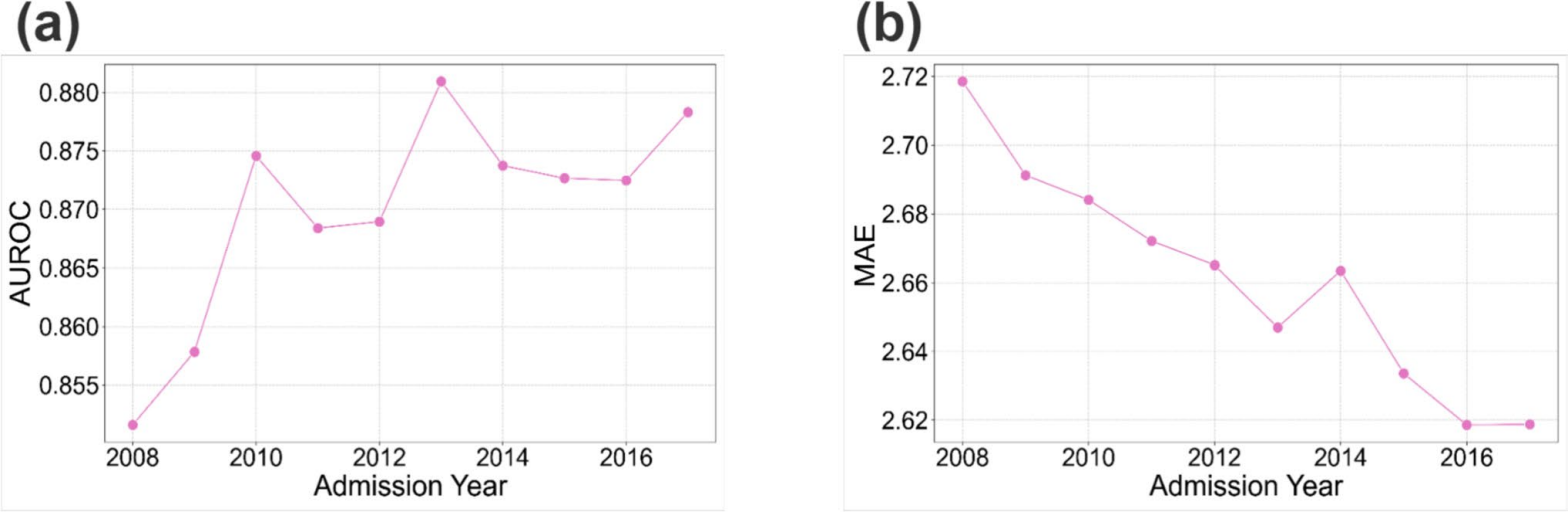
Performance of temporal validation. CatBoost was trained on datasets from different admission years, with results derived from the testing set for the year 2018. (**a**) displays AUROC for mortality prediction while (**b**) displays MAE for LOS prediction

We performed temporal validation to assess the model’s long-term stability against potential data drift. The CatBoost model was trained on data from each single admission year (2008 to 2017) and tested on a fixed 2018 dataset (Fig. 7). We used AUROC and MAE to evaluate performance for the two tasks, respectively. AUROC quantifies the model’s ability to discriminate between two classes across all possible decision thresholds, suitable for assessing CatBoost’s overall discriminative performance. For Task II, MAE was chosen because it is less sensitive to extreme outliers than MSE for the highly skewed LOS distribution (ranging from 0.1667 to 713.7403 days in this study).

For mortality prediction, the AUROC values remained above 0.85 across all 10 training years (Fig. 7(a)). This sustained performance, with the lowest AUROC being 0.8516 (trained on 2008 data), demonstrates that the fundamental predictive features maintained their core clinical utility against the temporal lag to 2018. Performance generally improved with more recent training data, but showed a non-monotonic trajectory: it peaked at an AUROC of 0.8809 (trained on 2013 data) before slightly declining to 0.8783 (trained on 2017 data). The 2013 peak suggests that the underlying feature space of this historical cohort was optimally generalizable to the 2018 clinical environment. However, when excluding this specific historical optimum, a clear pattern was observed where models trained on cohorts chronologically closer to 2018 generally exhibited superior performance compared to those trained on older data (2008–2012).

A more consistent trend was observed for LOS prediction, where the MAE steadily decreased as the training data became more recent. The MAE fell from 2.7186 (2008) to its lowest points of 2.6186 (2016) and 2.6188 (2017) (Fig. 7(b)). The observed inverse relationship between temporal distance and model performance underscores the critical importance of minimizing the temporal lag in training cohorts. These results indicate that retraining the model with newer data generally results in improved performance and is essential for maintaining optimal model accuracy in a dynamic clinical environment.

## Discussion

Predicting ICU mortality and LOS is crucial for guiding clinical decision-making, optimizing resource allocation, and improving patient outcomes [21, 36]. As elaborated in “Introduction” section, APACHE IV has shown limitations, notably its tendency to overestimate ICU mortality in Hong Kong between 2008 and 2018 [19]. While recent advancements in ML offer promising alternatives for outcome prediction, existing works [21, 28–36, 48–50] are hampered by limitations such as restricted dataset sizes, focus on specific populations, and a lack of comprehensive analyses (e.g., temporal validation, subgroup analysis).

This study contributes to this growing evidence by proposing an ML-based pipeline to predict ICU mortality and LOS using 11 years of data from 15 public hospitals in Hong Kong. Importantly, we emphasize both predictive accuracy and model interpretability, providing a practical foundation for future clinical integration.

### Principal Findings

Our results (Fig. 2, Table 3) show ML models’ substantial improvements in the two tasks compared to APACHE II and APACHE IV, which is consistent with prior studies demonstrating the superiority of ML models in healthcare applications [26, 28, 31, 34]. Among all models, CatBoost achieved the highest performance in both tasks, underscoring its capability to capture complex and non-linear relationships within high-dimensional data. Furthermore, ensemble ML models consistently outperformed linear models (LR and LASSO), a finding that aligns with previous studies [51–53], and reflects the limitations of linear models in handling non-linear feature interactions.

### Model Interpretability

Although ML models offer improved performance, their adoption in healthcare hinges on transparency. We addressed this by using SHAP methods to reveal the most influential predictors for the two tasks in “Feature Analysis” section, offering actionable guidance for clinical practice.

For mortality prediction, our analysis confirmed that older patients with low GCS and reduced Urine were at high risk of mortality, suggesting they warrant early, intensive intervention, in agreement with previous studies [54–56]. For LOS prediction, patients with high Creat (high) and abnormal respiratory rate measured on a ventilator tended to experience prolonged ICU stays. Notably, Alb (low) emerged as a top four influential feature for both mortality and LOS prediction. This indicates that patients with low Alb (low) are at a high risk of mortality and may require extended ICU stays for recovery, suggesting a critical need for focused attention. These indicators could support clinical decision-making, allowing physicians to closely monitor high-risk patients and optimize treatment strategies.

### Model Generalizability

Beyond overall performance, comprehending the extent and patterns of our models’ generalizability and identifying potential sources of performance variation across diverse clinical contexts was a key objective. To this end, we conducted subgroup analysis and temporal validation.

Subgroup analysis illustrated performance variations across patient characteristics and institutional settings in “Subgroup Performance” section. We specifically conducted an age group analysis on CatBoost to explore the effect of this important feature identified by SHAP. CatBoost performed best in younger age groups (Fig. 6(a) and (d)), likely owing to their more homogeneous clinical presentations, whereas older cohorts present complex comorbidities and heterogeneous conditions. This suggests that future research might benefit from age-specific modeling, particularly for older cohorts where comorbidities introduce greater variability.

In the hospital-specific evaluation, CatBoost’s performance for Task I was slightly lower across all hospitals compared to the full dataset, suggesting the benefit of larger, aggregated training samples for Task I. In contrast, several hospitals (e.g., Hospitals G and A) outperformed the overall model for Task II (Fig. 6(b) and (e)), indicating that Task II may be more sensitive to hospital settings and patient flow dynamics.

Temporal validation in “Temporal Validation” section indicated that using more recent training data may improve model performance, highlighting the dynamic nature of ICU care. This finding underscores the importance of regularly retraining models with recent patient data to ensure adaptability to evolving clinical practices and treatment protocols.

### Limitations

Despite the promising findings, this study has several limitations. First, to enable direct comparisons with APACHE models, we used features limited to the first 24 hours of ICU admission, excluding temporal and richer clinical data. Second, our mortality prediction provides a binary classification (death vs. survival) rather than a risk score. This design choice, while offering a clear decision boundary, limits the model’s ability to discriminate granularly between low-risk and intermediate-risk cases. Third, while interpretability methods like SHAP provide crucial insights into feature importance—explaining why the model made a specific prediction—they cannot directly establish the biological or clinical reason for a patient’s outcome. The strong predictive performance confirms the model’s utility as a decision-support tool, but it is not a replacement for expert clinical judgment. Lastly, model performance could be further enhanced through advanced fine-tuning, and external validation across different healthcare settings to ensure broader generalizability and clinical applicability.

## Conclusion

This study proposed a ML-based pipeline for the prediction of ICU mortality and LOS. The ML models evaluated in this study, particularly CatBoost, exhibited superior performance compared to the benchmark APACHE systems. Additionally, the use of SHAP analysis has improved the interpretability of the model, offering valuable insights into feature importance and enhancing model transparency. The performance across various subgroups further suggests the model’s potential for generalizability and highlights opportunities for tailored modeling in specific clinical environments. Beyond supporting clinical applications in Hong Kong, the proposed pipeline also provides a transferable framework adaptable to local healthcare systems.

## Data Availability

No datasets were generated or analysed during the current study.
